# Somatostatin analogs in neuroendocrine tumors with Ki‐67 index of ≥10%

**DOI:** 10.1111/jne.70112

**Published:** 2025-11-15

**Authors:** Johanna Braegelmann, Annie Mathew, Boerge Schmidt, Hamza Kalisch, Wolfgang P. Fendler, Dagmar Führer‐Sakel, Harald Lahner

**Affiliations:** ^1^ Department of Endocrinology, Diabetes and Metabolism and Division of Laboratory Research University Hospital Essen Essen Germany; ^2^ Institute of Medical Informatics, Biometry and Epidemiology University Hospital Essen Essen Germany; ^3^ Department of Nuclear Medicine University Hospital Essen Essen Germany; ^4^ Institute for Artificial Intelligence in Medicine (IKIM) University Hospital Essen (AöR) Essen Germany

**Keywords:** Ki‐67 index ≥10%, neuroendocrine tumor G2, neuroendocrine tumor G3, somatostatin analogues in NET

## Abstract

Somatostatin analogs (SSAs) are an established first‐line therapy in intestinal and pancreatic neuroendocrine tumors (NETs). Based on Phase III studies, their use is recommended in NET with a Ki‐67 index of up to 10%. The effect of first‐line SSA therapy on differentiated NET with a Ki‐67 index ≥10% is poorly understood. This study aimed to investigate the outcomes of SSA therapy in differentiated NETs with a Ki‐67 index of ≥10%. A retrospective analysis of a prospectively created dataset of consecutive patients with NETs was performed. Patients with first‐line SSA monotherapy in advanced NET with a Ki‐67 index ≥10% were included. The study endpoints were progression‐free survival (PFS), overall survival (OS), and clinical benefit rate, defined as partial remission (PR) or stable disease (SD). Of 362 consecutive patients with a Ki‐67 index ≥10%, 67 received first‐line SSA therapy. The Ki‐67 index was 10–20% (G2) in 57 (85%) patients and >20% (G3) in 10 (15%). SD as the best response was reached in 40 (59.7%) patients and PR in 3 (4.5%), irrespective of the NET origin, time from the diagnosis, or somatostatin receptor‐based tracer uptake. The median PFS was 18 (95% confidence interval [CI], 5.7–30.3) months, and the median OS was 60 (95% CI, 38.2–81.8) months after the initiation of SSA therapy. Median PFS was significantly longer in patients with a Ki‐67 index of 10–20% (19 months; 95% CI, 6.2–31.8) compared to those with G3 NETs (6 months; 95% CI, 2.9–9.1; *p* = .015, log‐rank test), and in patients with a liver tumor burden of ≤10% (24 months; 95% CI, 12.7–35.3) versus >10% (4 months; 95% CI, 2.3–5.7; *p* = .007). First‐line SSA therapy can provide meaningful disease control in patients with G2 NETs and low tumor burden, despite a Ki‐67 index ≥10%. It may be a reasonable alternative to more intensive therapies in selected patients.

## 1 INTRODUCTION

Two Phase III studies proposed long‐acting somatostatin analogs (SSAs) as the first‐line treatment for advanced gastroenteropancreatic (GEP) neuroendocrine tumors (NETs). In the double‐blind, placebo‐controlled PROMID trial, 85 patients diagnosed with differentiated metastatic midgut NETs were randomized to receive either long‐acting repeatable (LAR) 30 mg octreotide every 28 days or placebo.^1^ A significant improvement in the median time to progression was reported, from 6 months in the placebo group to 14.3 months in the SSA group (hazard ratio [HR], 0.34; *p* < .001). This marked a turning point in the treatment of NETs. However, the study considered virtually exclusively patients with G1 tumors, and the proliferation marker Ki‐67 was ≤2% in 95% of the cases. Subsequently, the double‐blind, placebo‐controlled CLARINET trial randomized 204 patients with metastatic GEP‐NETs with Ki‐67 index <10% to lanreotide depot 120 mg every 4 weeks or placebo.^2^ After a median study drug exposure of 24 months, the median progression‐free survival (PFS) was not achieved in the lanreotide group compared with 18 months in the placebo group (HR, 0.47; *p* < .001). These results were groundbreaking for the treatment of NETs with a Ki‐67 index up to 10%; however, the effectiveness of SSA in NETs with Ki‐67 index ≥10% could not be confirmed.^2^

In 2015, a study reported the antitumor effects of long‐acting lanreotide and octreotide according to the Ki‐67 index in patients with NET.^3^ The patients corresponded to all three grading classes according to the World Health Organization (WHO; G1 = Ki‐67 index <3%; G2 = Ki‐67 index 3–20%; G3 = Ki‐67 index >20%). Although the study, unlike PROMID and CLARINET, included tumors with a Ki‐67 index ≥10%, the focus was on G1 and G2 tumors with a low Ki‐67 index. The study showed a better tumor response in NETs with a Ki‐67 index <5%. No statement was made about the role of SSAs in G2 with a Ki‐67 ≥10% and well‐differentiated G3 tumors.^3^

Although SSAs play a pivotal role in the treatment of GEP‐NETs, evidence of their efficacy in NETs with Ki‐67 ≥10% remains limited. Retrospective studies have focused on pancreatic NETs only or on neuroendocrine neoplasia with higher proliferation (G3) treated with SSAs.^4^, ^5^, ^6^, ^7^

Most recently, the NETTER‐2 study investigated the use of peptide radioreceptor therapy (PRRT) as first‐line therapy in patients with G2 and G3 NETs (Ki‐67 index ≥10% to ≤55%).^8^ High‐dose SSA therapy (octreotide 60 mg LAR every 4 weeks) was chosen as the comparator arm; however, no standard of care has been established for these patients.

Therefore, this study aimed to evaluate the clinical outcomes of patients with NETs of various origins and Ki‐67 index ≥10% treated with standard‐dose, long‐acting SSAs as first‐line antitumor therapy.

## 2 PATIENTS AND METHODS

Patients with histologically confirmed, locally advanced or metastatic NET with Ki‐67 index ≥10%, who received SSA monotherapy as first‐line drug therapy, were identified from our prospective database at the European Neuroendocrine Tumor Society Center of Excellence at the University Hospital of Essen. Cross‐sectional imaging (computed tomography [CT] or magnetic resonance imaging) was performed before the therapy commenced and during the disease course. Hepatic tumor burden was quantified and categorized for statistical analysis. Somatostatin receptor (SSTR)‐based hybrid imaging with contrast‐enhanced CT (^68^Ga DOTATOC positron emission tomography [PET]/ceCT) was included at the initial presentation and at least every 12 months within the surveillance schedule. To assess the clinical benefit, the progression status of the first staging after starting SSA therapy was evaluated. Both functionally active and inactive NETs were included. Patients who received PRRT parallel to their SSA therapy were excluded. The primary endpoint was PFS, and the secondary endpoints were overall survival (OS) and response to therapy.

### 2.1 Statistical analysis

Categorical data are reported as numbers and percentages within the cohort. For quantitative data, the median, unless otherwise stated, was determined. PFS was defined as the time between the start of therapy and disease progression according to the response evaluation criteria in solid tumors, version 1.1 (RECIST 1.1) criteria or death.^9^ The time between the start of SSA therapy and death from any cause was defined as OS. PFS and OS were estimated using the Kaplan–Meier method and compared by the log‐rank test. Results with a *p*‐value of <.05 (two‐sided) were considered significant. All statistical analyses were performed using the IBM SPSS Statistics version 26.0 (IBM Corp., Armonk, NY, USA).

Written informed patient consent and approval for data collection and analysis were obtained upon admission to our institution. The study was approved by the local ethics committee (18‐8367‐BO).

## 3 RESULTS

In this study, 362 consecutive patients with advanced, differentiated NETs and Ki‐67 index ≥10% presented to our center for the first time between January 2010 and February 2024. Of these, 67 (18.5%) patients received first‐line drug therapy with long‐acting SSAs according to the recommendation of the interdisciplinary tumor board (ITB) based on oligometastatic, low tumor burden, and low growth rate. Forty‐four patients underwent primary surgery without cure. This was mainly due to extensive lymphonodular involvement, mesenteric fibrosis or, in the case of pancreatic NETs, involvement of surrounding vessels. In the remaining 23 patients, the ITB recommended initial treatment with SSA, as surgery was not considered promising. Of the total cohort, five patients had disseminated lymph node metastases only and 46 patients had distant liver metastases only. Eleven patients had liver and bone metastases and five patients had metastases in more than two organ systems. The majority of patients (56.7%) had a liver tumor burden of less than or equal to 10% at the start of SSA therapy, 9.0% had a liver tumor burden between greater than 10% and 25%, and 7.5% had a liver tumor burden greater than 25%. In 26.9% of the patients, the liver tumor burden could not be determined, because not all cross‐sectional images were available for evaluation. All patients were evaluable for PFS, OS, and response to therapy. The median age at the first diagnosis was 58 (range 33–84) years, and approximately the same number of men (*n* = 34) and women (*n* = 33) was included. The most common primary localization was the pancreas (*n* = 22), followed by small intestinal NET (*n* = 18), cancer of unknown primary (CUP; *n* = 9), and lungs (*n* = 7). The remaining primary locations were subsumed into the group “other” (cecum, *n* = 4; stomach, *n* = 3; ascending colon, *n* = 2; rectum, *n* = 1; parotid gland, *n* = 1). Most patients (57/67) had a G2 tumor with a median Ki‐67 index of 15% (95% CI, 10–15%). In contrast, 10 patients had a G3 differentiated NET with a median Ki‐67 index of 30% (95% CI, 25–40%). Pathological analyses were performed on biopsy and surgical samples in 41 and 26 cases, respectively. All patients had somatostatin receptor expression in all target lesions as assessed by hybrid imaging (^68^Ga DOTATOC PET/CT). Somatostatin receptor uptake was scored using a visual semiquantitative scale.^10^ Sixty percent of patients had an uptake score of 3 (greater than liver but lower than spleen), 40% had a score of 4 (greater than spleen). Approximately, the same number of patients received SSA therapy immediately after diagnosis (<3 months, *n* = 35) as 3 months after initial diagnosis or later (*n* = 32). In 20 (29.9%) patients, therapy was started more than 6 months after the initial diagnosis of NETs. In the subgroup of patients with late initiation of SSA therapy, radiographic progression prior to initiation was observed in 17 of 32 patients (53%). Serotonin excess was detected in 21 patients (31%) due to elevated 5‐hydroxyindoleacetic acid (5‐HIAA). No patient received above‐label dosages of SSA. The median frequency of follow‐up imaging during SSA therapy was every 4 (95% confidence interval [CI], 3–6) months. Baseline clinicopathological characteristics are summarized in Table 1.

**TABLE 1:** Demographic and baseline clinical characteristics of the patients (*n* = 67).

Characteristic	*n* (%)
Age at initial diagnosis, years	
Median	58
Range	33–84
Sex	
Male	34 (50.7)
Female	33 (49.3)
Origin of NET	
Pancreas	22 (32.8)
Lung	7 (10.4)
Small intestine	18 (26.9)
Cancer of unknown primary (CUP)	9 (13.4)
Other	11 (16.4)
Functional status	
Functioning (5‐HIAA elevated)	21 (31.3)
Non‐functioning	46 (68.7)
Stage IV at start of SSA therapy	
No (lymph node involvement only)	5 (7.5)
Yes	62 (92.5)
*Liver metastases*	*46* (*68.7*)
*Liver and Bone metastases*	*11* (*16.4*)
*Metastases in more than 2 organ systems*	*5* (*7.5*)
Hepatic tumor load	
0–10%	38 (56.7)
>10–25%	6 (9.0)
>25–50%	3 (4.5)
>50%	2 (3.0)
Unknown	18 (26.9)
Ki‐67 index at initial diagnosis	
10–20%	57 (85.1)
>20%	10 (14.9)
Time from initial diagnosis to start of SSA therapy	
<3 months	35 (52.2)
≥3 months	32 (47.8)
>6 months	20 (29.9)
Somatostatin‐analogue	
Lanreotide	47 (70.1)
Octreotide	20 (29.9)
Increased tracer uptake on ^68^Ga DOTATOC PET/CT	
Partial	40 (59.7)
Total	27 (40.3)

*Note*: Values are given as n (%) unless otherwise indicated.

Abbreviations: CT, computed tomography; NET, neuroendocrine tumor; PET, positron emission tomography; SSA, somatostatin analog; 5‐HIAA, 5‐hydroxyindoleacetic acid.

Of the 67 patients, 43 showed either stable disease (SD, 40/67, 59.7%) or partial remission (PR, 3/67, 4.5%) in the first imaging after starting the SSA therapy, accounting for a clinical benefit rate (CBR, SD + PR) of 64.2%. Progressive disease (PD) was observed in 24/67 (35.8%) patients. A liver tumor burden of ≤10% was associated with better disease control (SD + PR vs. PD, odds ratio [OR] 12.6, 95% CI 2.3–62.0, *p* = .0015; Table 2). Treatment response was stable across all other subgroups in terms of primary tumor (pancreatic vs. extrapancreatic), timing of SSA therapy initiation (<3 vs. ≥3 months after diagnosis), progression status at therapy initiation, functional status, and SSTR tracer uptake score. Patients with G2 NETs tended to have a greater CBR than patients with G3 NET (OR 3.3, 95% CI 0.9–11.0, *p* = n.s.).

**TABLE 2:** Clinical benefit of SSA therapy (Fisher's exact test).

	Clinical benefit (SD, PR), *n*	Progressive disease (PD), *n*	*p*
All patients	43 (64.2%)	24 (35.8%)	
Origin of NET			
Pancreatic	14	8	
Extrapancreatic	29	16	*>.9 (n.s.)*
Grading			
G2	39	18	
G3	4	6	.*15 (n.s.)*
Start of therapy			
<3 months	23	12	
≥3 months	20	12	.*8 (n.s.)*
Increased tracer uptake			
Partial	26	14	
Total	17	10	>.*9 (n.s.)*
Hepatic tumor load^a^			.*0015*
≤10%	28	10	
>10%	2	9	

^a^ Evaluable in n = 49 subjects.

Abbreviations: NET, neuroendocrine tumor; PR, partial remission; SD, stable disease; SSA, somatostatin analog, n.s., not significant.

**FIGURE 1:**
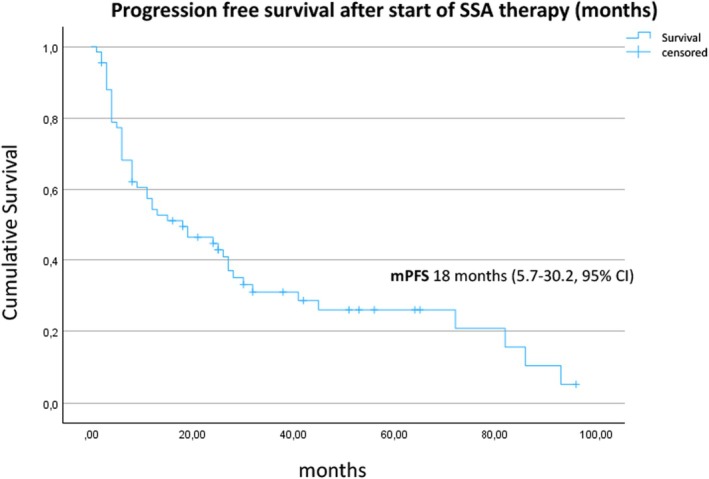
Kaplan–Meier estimate of the survival function for the progression‐free survival after the start of SSA therapy for all 67 patients. SSA, somatostatin analog.

**FIGURE 2:**
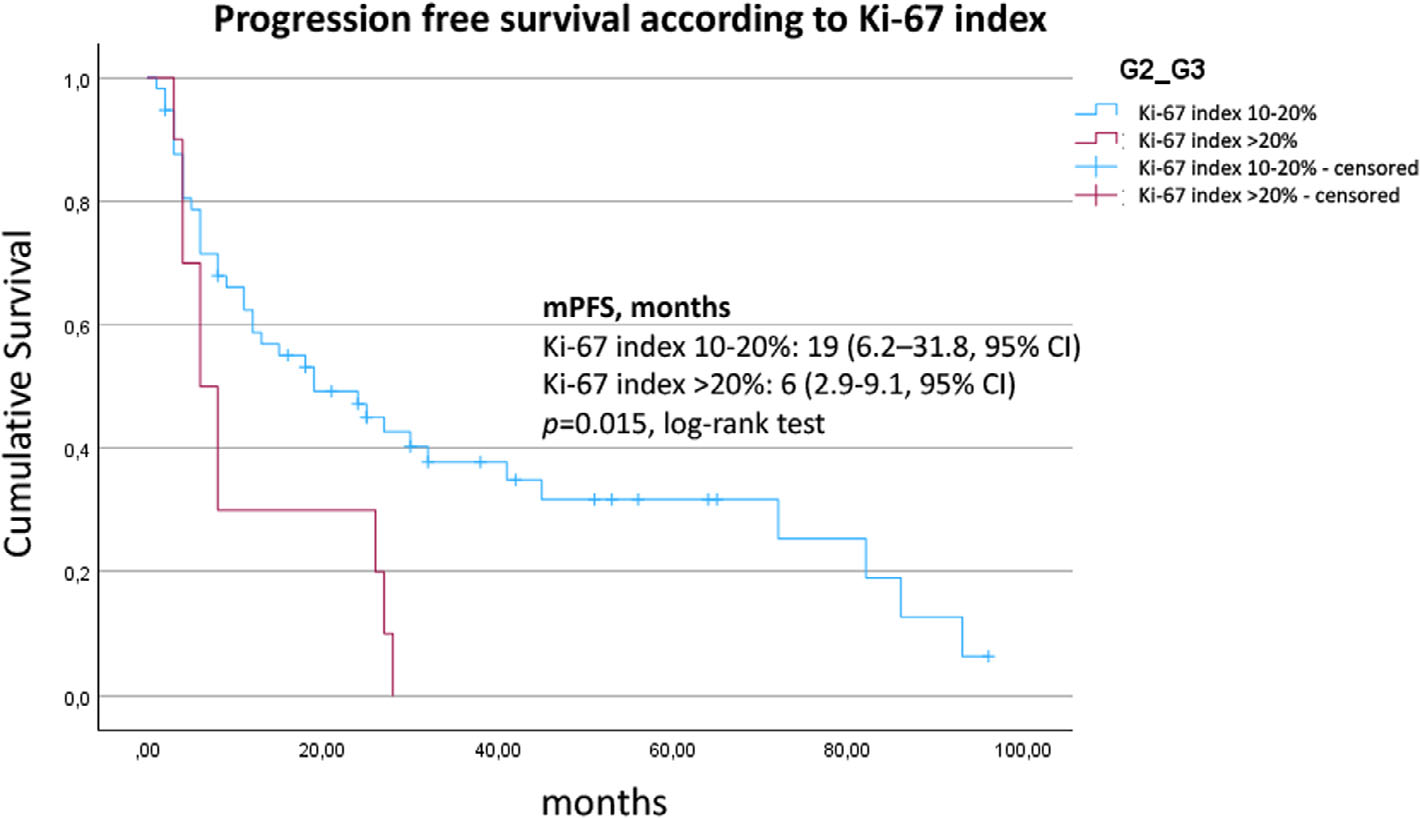
Kaplan–Meier estimate of the survival function for the progression‐free survival, stratified by Ki‐67 proliferation marker.

**FIGURE 3:**
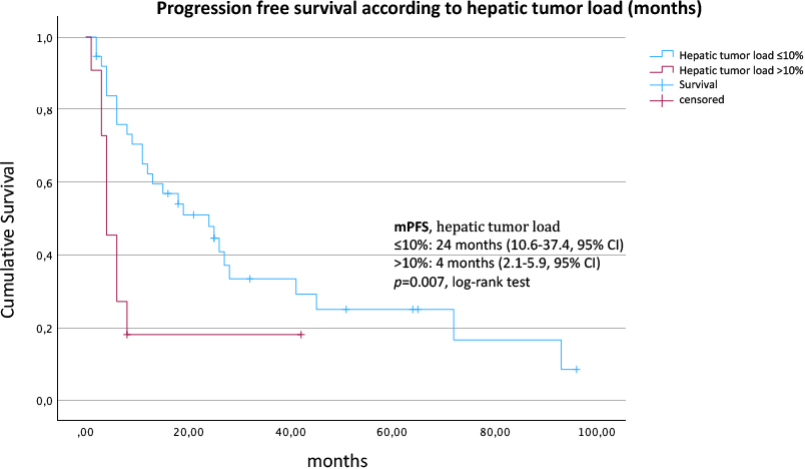
Kaplan–Meier estimate of the survival function for the progression‐free survival according to hepatic tumor load (≤10% vs. >10%).

**FIGURE 4:**
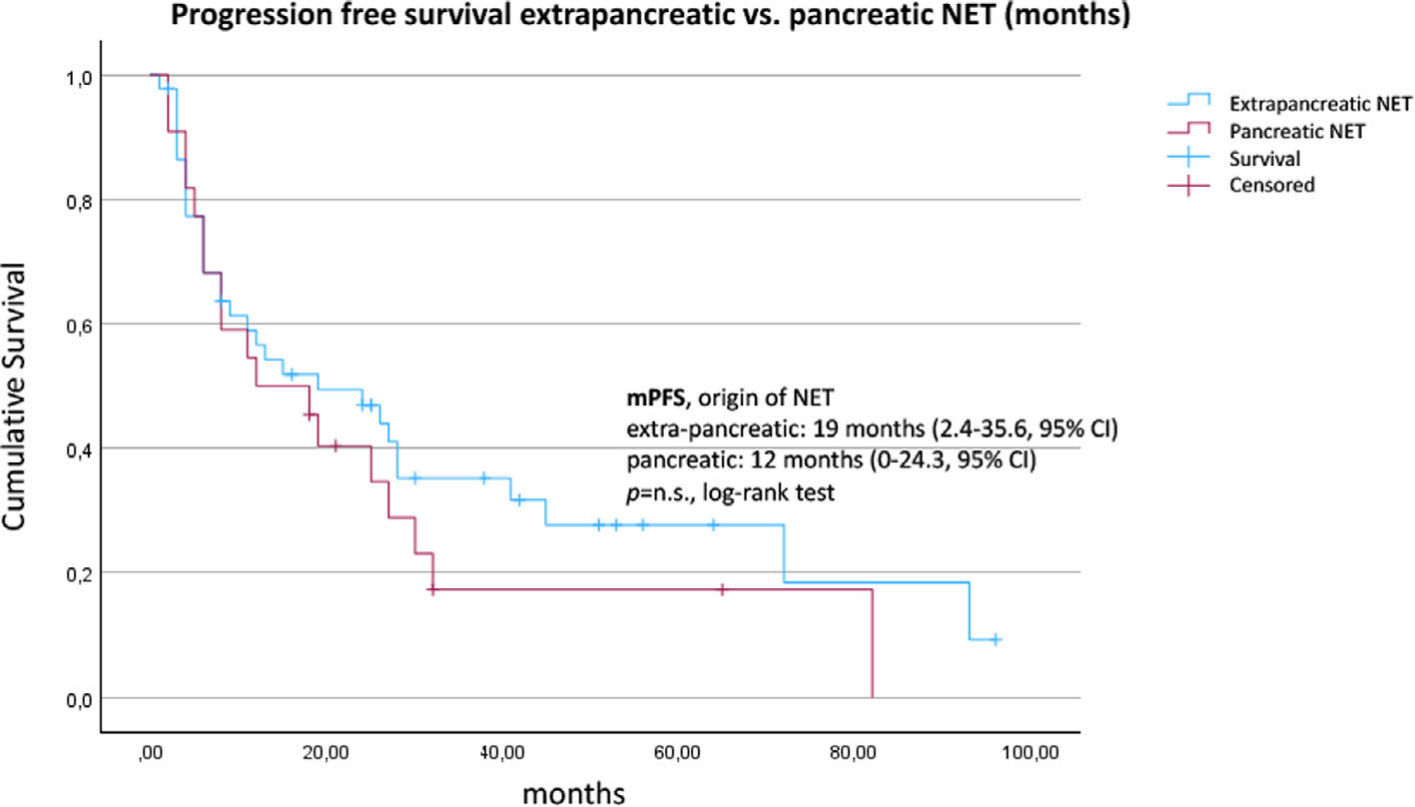
Kaplan–Meier estimate of the survival function for the progression‐free survival according to the origin of NET (extrapancreatic vs. pancreatic). NET, neuroendocrine tumor.

At the time of data analysis, 49/67 (73.1%) patients had progressed, whereas 18 patients were still on treatment. The median PFS from the initiation of SSA therapy to progression according to RECIST criteria was 18 (95% CI, 5.7–30.2) months (Figure 1). The median PFS was 19 (95% CI, 6.2–31.8) months for patients with a Ki‐67 index between 10% and 20% in comparison with 6 (95% CI, 2.9–9.1) months for patients with NET G3 (Ki‐67 index >20%) (*p* = .015, Figure 2). Liver burden also had a significant association with PFS. Patients with a tumor burden <10% had a median PFS of 24 (95% CI, 10.6–37.4) months compared to 4 (95% CI, 3.8–22.2) months for patients with a higher burden (*p* = .007, log‐rank test) (Figure 3). Stratified by the NET origin, a trend was noted towards a longer median PFS of 24 months for small intestinal NETs and 26 months for NETs of other locations (“others”), whereas pulmonary (8 months), pancreatic (12 months), and CUP (13 months) NETs revealed worse outcomes, without reaching significance (Figure 4). No difference in PFS was found according to somatostatin receptor uptake score (3 vs. 4) and administered SSAs (octreotide vs. lanreotide).

**FIGURE 5:**
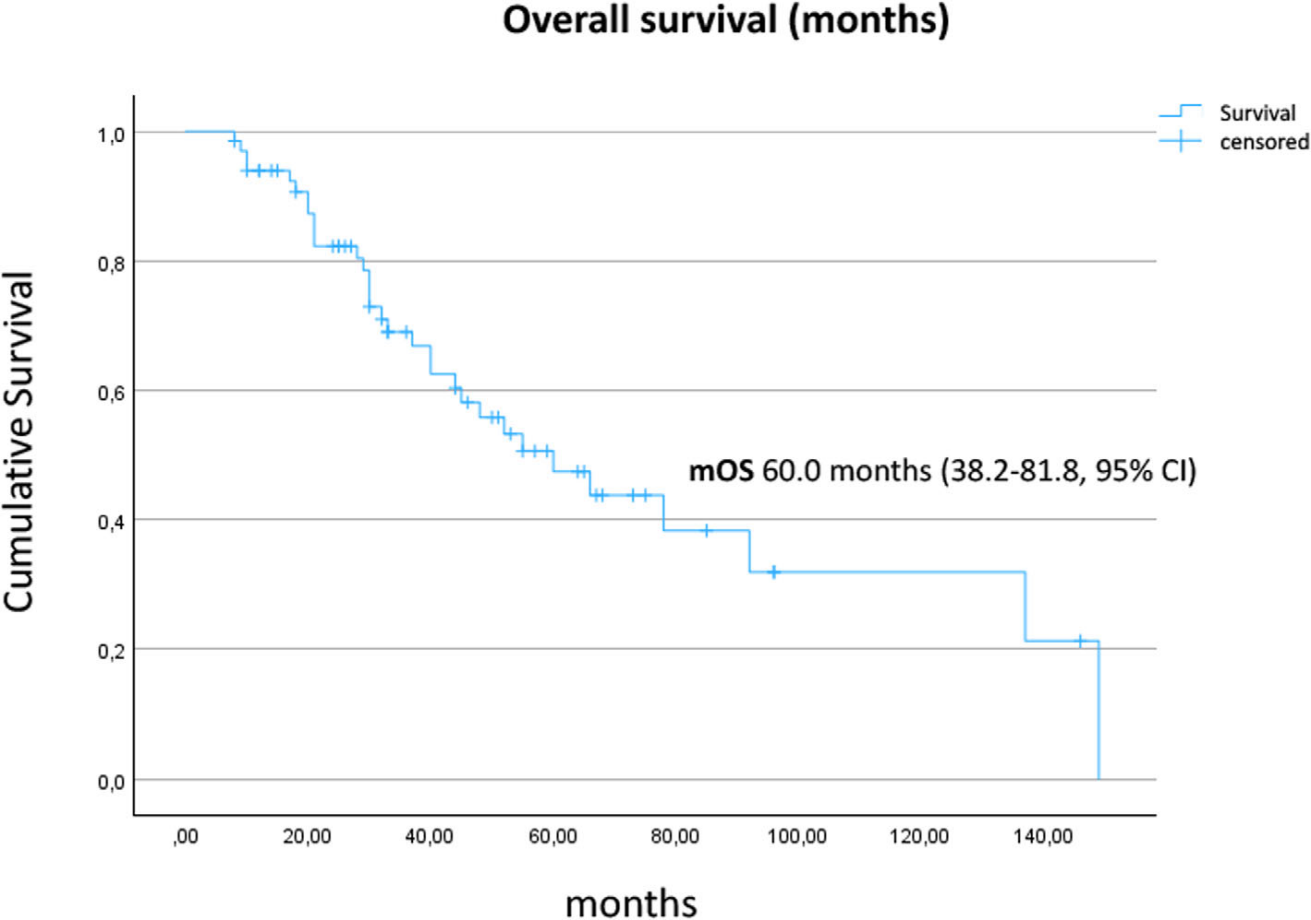
Kaplan–Meier estimate of the overall survival after start of SSA therapy for all 67 patients. SSA, somatostatin analog.

At the data cutoff, 32 patients had died. The median OS after starting SSA therapy was 60.0 (95% CI, 38.2–81.8) months (Figure 5). The median OS was 66.0 (95% CI, 28.0–104.0) months in patients with a Ki‐67 index between 10% and 20% and 40.0 (95% CI, 27.0–53.0 months, *p* = n.s., log‐rank test) in patients with G3 tumors. Liver burden >10% was associated with a trend toward shorter median OS (30 months, 95% CI, 4.6–55.4) compared to patients with lower liver burden (60 months, 95% CI, 31.8–88.2; *p* = n.s., log‐rank test). The functionality (5‐HIAA elevated vs. not), SSTR uptake score (3 vs. 4), and administered SSA (octreotide vs. lanreotide) did not show any prognostic effect.^11^ The median OS after the initial diagnosis of NET was 80.0 (95% CI, 47.8–112.2) months.

## 4 DISCUSSION

In our cohort of 67 carefully selected patients with advanced, differentiated NETs with an increased proliferation rate, the first‐line treatment with SSA was associated with a disease stabilization rate of 64.2%, median PFS of 18 (95% CI, 5.7–30.2) months, and median OS of 60 (95% CI, 38.2–81.8) months. A low liver tumor burden of ≤10% was associated with a higher rate of disease stabilization (OR 12.6, *p* = .0015) and a significantly longer median PFS (24 vs. 4 months, *p* = .007). SSA therapy in NETs with a Ki‐67 index between 10% and 20% was associated with a significantly longer median PFS compared to G3 NETs (19 vs. 6 months, *p* = .015).

Selection bias in favor of a more favorable disease course in our cohort is likely. Patients with metastases in a maximum of two organ systems, a liver tumor burden equal to or less than 10% and a Ki‐67 proliferation index between 10% and 15% were overrepresented. Only 18.5% of all NET patients with a Ki‐67 index ≥10% presenting to our institution were recommended for SSA first‐line therapy by the ITB. In this context, a remarkable proportion of patients (31% of the cohort) showed serotonin production by the tumor. Functionally active NET may be diagnosed earlier in the course of the disease, when the tumor volume is still small, compared to the hormonally inactive forms due to their clinical symptoms. Patients with rapid progression or high tumor burden as assessed by the ITB received more intensive treatment such as PRRT, everolimus or streptozocin‐based chemotherapy. They were not a priori candidates for the SSA therapy studied here.

Recently, the NETTER‐2 trial examined the effect of four cycles of intravenous ^177^Lu‐Dotatate (PRRT) plus continued intramuscular 30 mg LAR octreotide every 4 weeks versus high‐dose SSA (octreotide 60 mg every 4 weeks).^8^ Patients with newly diagnosed higher Grade 2 (Ki‐67 index ≥10% and ≤ 20%) and Grade 3 (Ki‐67 index >20% and ≤ 55%), SSTR‐positive, advanced GEP‐NETs were included. Patients were randomly assigned (2:1) to receive PRRT plus octreotide 30 mg LAR or high‐dose octreotide 60 mg LAR (control group). The median PFS was 8.5 (95% CI, 7.7–13.8) and 22.8 months (19.4 to not estimated) in the control and ^177^Lu‐Dotatate (hazard ratio [HR] 0.276; *p* < .0001) groups, respectively.^8^ The disease control rates (DCRs) were 66.7% (95% CI, 54.8–77.1) and 90.7% (95% CI, 84.9–94.8) in the control and PRRT groups, respectively.

The inclusion criteria were largely consistent with those of the present study, with the difference that in NETTER‐2 patients were included within 6 months of initial diagnosis unselected in terms of tumor dynamics, whereas in the present study, an oligometastatic low tumor burden and—as far as assessable—slow tumor growth were prerequisites for treatment. In addition, NETTER‐2 enrolled more patients with G3 NET than this study (35% vs. 15%). The median PFS of our cohort was 18 months, clearly longer than the 8.5 months of the SSA high‐dose control group of the NETTER‐2 study and close to that of the PRRT intervention group. Moreover, the DCR of 64.2% in the present study was comparable to that of the NETTER‐2 study (66.7%), which used twice the SSA dose. The better results in our patients can be explained by the fact that we used SSA therapy according to clinical practice. As a result, despite the elevated Ki‐67 marker, we enrolled mainly patients with relatively slow progressing NET. A very similar observation was made by Lamberti et al. who retrospectively analyzed 140 patients with G1 and (low) G2 NET.^12^ The patients had received high‐dose SSA therapy after progression (q14/21d) and were therefore very similar to the control group of the NETTER‐1 trial. As in our study, the median PFS in the retrospective study was significantly higher (31.0 months) than in the prospective NETTER‐1 study (8.4 months).^13^ We conclude that G2 NET can also be treated with long‐acting SSA if they are oligometastatic, slowly progressing and have low tumor burden. The combination with PRRT can lead to additional prolongation of PFS and OS, but shows more hematotoxicity.^8^

Merola et al. focused on the effectiveness of SSA explicitly for pancreatic NETs with a Ki‐67 index ≥10%.^4^ The study included 73 patients across 10 centers, and 68 of them had G2 and 5 G3 NETs. The median time to next treatment was 14.2 (95% CI, 11.6–16.2) months, and the PFS was 11.9 (95% CI, 8.6–14.1) months. The median OS from diagnosis was 86 (95% CI, 56.8–86) months. The markedly longer PFS in our patients (18.0 vs. 11.9 months) may have several reasons. First, most NETs studied had an extrapancreatic origin, which may have different outcomes.^14^ Second, the median tumor burden in the liver was markedly higher in the Merola study. Only a quarter (27%) of the patients had a liver tumor burden of ≤10%, compared to 77.6% of those we studied. Moreover, the Merola study showed that liver involvement was a strong predictor of outcome. Using the cutoff of 25% for liver tumor burden, the median PFS of 15 (95% CI, 12.2–19.8) months was significantly higher in this study and well in line with our results. Our observation that a longer PFS was practically only noted in the G2 NET group is in line with the results of Merola et al. The median OS from the first diagnosis, 80 months in this study compared with 86 months in the study by Merola et al., correlated in both studies. In summary, patients with oligometastatic NETs and low hepatic tumor burden may have a PFS of 15–18 months associated with the SSA first‐line therapy, even with higher proliferative G2 NETs.

In 2015, Faggiano et al. aimed to investigate the antitumor effects of long‐acting SSAs in 140 patients with NETs according to the Ki67 index.^3^ In this study, a surprisingly long PFS was reported, which did not differ significantly between G1 and G2 NETs (median, 89 vs. 43 months, *p* = .15). However, the median PFS was significantly longer in NETs showing Ki67 < 5% than in those showing ≥5% (89 vs. 35 months, *p* = .005). The most likely reason is that, in contrast to the present study, about half of the patients had localized disease, and only 45% had metastatic, Union for International Cancer Control Stage IV NETs. In addition, some patients were treated with a second antiproliferative therapy simultaneously. In this respect, the cohort differed significantly from the present study of first‐line SSA therapy in advanced, systemic metastatic NETs. The strength of the work of Faggiano et al. lies in the identification of the Ki‐67 index cutoff of 5%, up to which SSA therapy was associated with better efficacy.^3^ However, the role of SSAs as first‐line monotherapy in higher proliferative G2 NETs was not assessed.

In recent years, several retrospective studies have focused on the treatment options for differentiated G3 NETs. In this context, three authors reported that SSA therapy was associated with a median PFS of 4–8 months. In the most comprehensive study by de Mestier et al., 19 patients received first‐line SSA therapy.^6^ The median PFS in this group was 6.2 (95% CI, 3.9–8.4) months, the CBR was 75% (best response), and the OS was not reported. The authors conclude that the use of SSAs plays a limited role in differentiated G3 NETs but can be considered in individual cases due to the favorable toxicity profile. We confirm this judgment. In contrast to G2 NETs, SSA therapy for G3 NETs in the present study was associated with a significantly shorter PFS (6 vs. 19 months, *p* = .015) and limited CBR (4/10, best response), even with the initial selection of patients with oligometastasis. However, our conclusions about this subgroup are limited by the relatively small sample size for G3 NETs (*n* = 10). While the results in G2 NETs are promising, the applicability to G3 NETs remains unclear. SSA therapy for G3 NETs is not supported by the data. Boutin et al. analyzed patients with differentiated G3 NETs using a regional database.^7^ Of a total of 41 patients, 14 received SSA first‐line therapy with a PFS of 7.9 months, with no OS indication. However, a statistical bias is possible because the PFS was calculated from the clinical documentation in the database if this indicated a change in therapy. In the absence of an observation interval, the 7.9 months are more likely to be interpreted as the time until the next line of treatment. Lithgow et al. examined 26 patients with G3 NET, and 9 of whom received SSA therapy and 3 of whom showed stable disease as the best response, with a median PFS of 4 months.^5^ The authors conclude that SSA therapy in G3 NETs is an option for selected patients.

The present study is limited by its retrospective design. In particular, the selection of patients to receive SSA therapy was made individually by an ITB. We took this into account by evaluating the ITB selection criteria and quantifying the clinical impression of oligometastatic, slow‐growing NET. Nevertheless, a statistically skewed distribution of the cohort with a trend toward less aggressive tumor growth can be assumed. Therefore, the patients studied do not constitute a representative sample of higher proliferating NETs. In addition, given the small number of patients with G3 tumors, the statistical power for subgroup analysis is low. Conversely, one of the major strengths of the study is the systematic, prospective follow‐up of patients. To optimize tailored therapeutic strategies for this specific patient population, controlled trials are needed to validate these results.

## 5 CONCLUSION

Long‐acting SSAs are a treatment option for selected G2 NET patients with a liver tumor burden of <10% and metastases in a maximum of two organ systems. They can be used in the first‐line setting instead of other more toxic treatments.

## AUTHOR CONTRIBUTIONS

**Johanna Braegelmann:** Conceptualization; investigation; writing – original draft; methodology; formal analysis; project administration; data curation; software. **Annie Mathew:** Writing – review and editing; methodology; formal analysis; supervision. **Boerge Schmidt:** Writing – review and editing; methodology; formal analysis. **Hamza Kalisch:** Validation; methodology; software; data curation. **Wolfgang P. Fendler:** Formal analysis; software. **Dagmar Führer‐Sakel:** Writing – review and editing; supervision. **Harald Lahner:** Investigation; writing – original draft; writing – review and editing; formal analysis; supervision; conceptualization; validation; visualization; software; project administration; data curation.

## FUNDING INFORMATION

The authors did not receive financial support for the research, authorship and/or publication of this article.

## CONFLICT OF INTEREST STATEMENT

The authors declare no conflicts of interest.

## ETHICS STATEMENT

Written informed patient consent and approval for data collection and analysis were obtained upon admission to our institution. The study was approved by the local ethics committee (18‐8367‐BO).

## Data Availability

The data that support the findings of this study are available on request from the corresponding author. The data are not publicly available due to their containing information that could compromise the privacy of research participants.
